# *catena*-Poly[[chloridocopper(II)]bis­(μ-3,3′,5,5′-tetra­methyl-4,4′-methylene­dipyrazole)[chloridocopper(II)]-di-μ-chlorido]

**DOI:** 10.1107/S1600536808006909

**Published:** 2008-03-14

**Authors:** Zhi-Min Wang

**Affiliations:** a College of Biology and Environmental Engineering, Zhejiang shuren University, Hangzhou 310015, People’s Republic of China

## Abstract

In the title compound, [Cu_2_Cl_4_(C_11_H_16_N_4_)]_*n*_, the Cu atom is coordinated by two N atoms of two 3,3′,5,5′-tetra­methyl-4,4′-methyl­enedipyrazole (H_2_mbdpz) ligands, two bridging Cl atoms and one terminal Cl atom, forming a square-pyramidal geometry. The bridging Cl atoms and the bridging H_2_mbdpz ligands connect the Cu atoms to build up an extended one-dimensional chain. The chains are further connected through N—H⋯Cl hydrogen bonds to build up a two-dimensional layer in the (011) plane. An inversion centre lies between every pair of adjacent Cu atoms.

## Related literature

For related literature, see: Kaes *et al.* (1998 ▶); Yaghi *et al.* (1998 ▶); Yagi *et al.* (2002 ▶); Nassimbeni (2003 ▶).
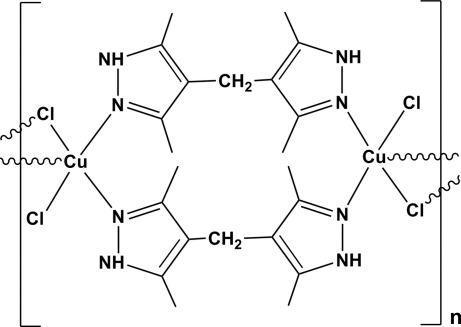


## Experimental

### 

#### Crystal data

[Cu_2_Cl_4_(C_11_H_16_N_4_)]
*M* *_r_* = 677.44
Triclinic, 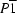

*a* = 8.759 (3) Å
*b* = 8.879 (3) Å
*c* = 9.735 (3) Å
α = 79.269 (6)°
β = 63.584 (5)°
γ = 86.922 (5)°
*V* = 665.8 (4) Å^3^
*Z* = 1
Mo *K*α radiation
μ = 2.03 mm^−1^
*T* = 298 (2) K
0.26 × 0.23 × 0.19 mm

#### Data collection

Bruker APEXII area-detector diffractometer
Absorption correction: multi-scan (*SADABS*; Sheldrick, 2004 ▶) *T*_min_ = 0.621, *T*_max_ = 0.699
3354 measured reflections
2331 independent reflections
1541 reflections with *I* > 2σ(*I*)
*R*_int_ = 0.033

#### Refinement

*R*[*F* ^2^ > 2σ(*F* ^2^)] = 0.064
*wR*(*F* ^2^) = 0.178
*S* = 0.99
2331 reflections
167 parameters
H-atom parameters constrained
Δρ_max_ = 0.79 e Å^−3^
Δρ_min_ = −1.14 e Å^−3^

### 

Data collection: *APEX2* (Bruker, 2004 ▶); cell refinement: *SAINT* (Bruker, 2004 ▶); data reduction: *SAINT*; program(s) used to solve structure: *SHELXS97* (Sheldrick, 2008 ▶); program(s) used to refine structure: *SHELXL97* (Sheldrick, 2008 ▶); molecular graphics: *PLATON* (Spek, 2003 ▶); software used to prepare material for publication: *SHELXL97*.

## Supplementary Material

Crystal structure: contains datablocks I, global. DOI: 10.1107/S1600536808006909/dn2323sup1.cif

Structure factors: contains datablocks I. DOI: 10.1107/S1600536808006909/dn2323Isup2.hkl

Additional supplementary materials:  crystallographic information; 3D view; checkCIF report

## Figures and Tables

**Table 1:** Hydrogen-bond geometry (Å, °)

*D*—H⋯*A*	*D*—H	H⋯*A*	*D*⋯*A*	*D*—H⋯*A*
N2—H2⋯Cl1^i^	0.86	2.43	3.242 (6)	157
N4—H4⋯Cl1^ii^	0.86	2.34	3.172 (6)	164

Symmetry codes: (i) 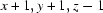; (ii) 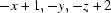.

**Fig. 1.**
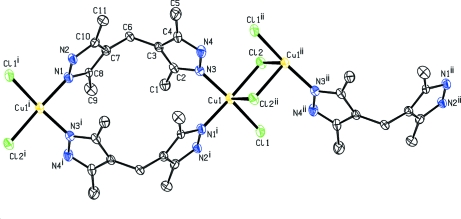
View of compound (I) with the atom-labelling scheme. Ellipsoids are drawn at the 30% probability level. H atoms have been omitted for clarity. [Symmetry code: (i) 1 - x, 1 - y, 1 - z; (ii) 1 - x, -y, 2 - z].

## supplementary crystallographic information

### Comment

Considerable research efforts have been devoted to searching for new and better inclusion compounds. One of the main reasons is their potential for eventual applications in a variety of technologically useful processes (Nassimbeni, 2003). In the past performance, the majority of cases in one-dimensional coordination networks was focused on bis-monodentate ligand (Yaghi *et al.*, 1998), while a few examples of bis-bidentate, bis-tridentate ones were documented(Kaes *et al.*, 1998). Here, we reported a 1-D complexes using the bis-bidentate ligand 4,4'-methylene-bis(3,5-dimethylpyrazole) (H_2_mbdpz).

In the title compound (I), the copper atom is coordinated by two nitrogen atoms of the H_2_mbdpz ligand, two bridging chlorine atom and one terminal chlorine, forming a square-pyramidal geometry (Fig. 1). The average Cu—N bond lengths, 1.999 (3) Å, is longer than those observed in other copper complexes (Yagi *et al.*, 2002). The average Cu—Cl bond lengths is 2.439 (3) Å. the bridging chlorine atoms and the bridging H_2_mbdpz ligands connect the copper atoms to build up an extended one dimensionnal chain (Fig. 1). The chains are further connected through N—H···Cl hydrogen bonds to build up a two-dimensionnal layer along the (0 1 1) plane (Table 1).

### Experimental

CuCl_2_(0.028 g, 0.015 mmol), H_2_mbdpz(0.023 g, 0.012 mmol) were added to methanol. The mixture was heated for ten hours under reflux. The resultant was then filtered to give a pure solution. Two weeks later suitable single crystals for X-Ray diffraction analysis were obtained.

### Refinement

All H atoms attached to C and N atom were fixed geometrically and treated as riding with C—H = 0.96 Å (methyl), 0.97 Å (methylene) and N—H = 0.86 Å with *U*_iso_(H) = 1.2*U*_eq_(C, N) or *U*_iso_(H) = 1.5*U*_eq_(methyl).

### Crystal data

**Table:**

[Cu_2_Cl_4_(C_11_H_16_N_4_)]	*Z* = 1
*M**_r_* = 677.44	*F*_000_ = 346
Triclinic, *P*1	*D*_x_ = 1.689 Mg m^−^^3^
Hall symbol: -P 1	Mo *K*α radiation λ = 0.71073 Å
*a* = 8.759 (3) Å	Cell parameters from 2334 reflections
*b* = 8.879 (3) Å	θ = 2.3–25.2º
*c* = 9.735 (3) Å	µ = 2.03 mm^−^^1^
α = 79.269 (6)º	*T* = 298 (2) K
β = 63.584 (5)º	Block, blue
γ = 86.922 (5)º	0.26 × 0.23 × 0.19 mm
*V* = 665.8 (4) Å^3^	

### Data collection

**Table:**

Bruker APEXII area-detector diffractometer	2331 independent reflections
Radiation source: fine-focus sealed tube	1541 reflections with *I* > 2σ(*I*)
Monochromator: graphite	*R*_int_ = 0.033
*T* = 298(2) K	θ_max_ = 25.2º
φ and ω scans	θ_min_ = 2.3º
Absorption correction: multi-scan(SADABS; Sheldrick, 2004)	*h* = −10→9
*T*_min_ = 0.621, *T*_max_ = 0.699	*k* = −7→10
3354 measured reflections	*l* = −11→11

### Refinement

**Table:**

Refinement on *F*^2^	Secondary atom site location: difference Fourier map
Least-squares matrix: full	Hydrogen site location: inferred from neighbouring sites
*R*[*F*^2^ > 2σ(*F*^2^)] = 0.064	H-atom parameters constrained
*wR*(*F*^2^) = 0.178	*w* = 1/[σ^2^(*F*_o_^2^) + (0.1072*P*)^2^] where *P* = (*F*_o_^2^ + 2*F*_c_^2^)/3
*S* = 0.99	(Δ/σ)_max_ = 0.001
2331 reflections	Δρ_max_ = 0.79 e Å^−^^3^
167 parameters	Δρ_min_ = −1.14 e Å^−^^3^
Primary atom site location: structure-invariant direct methods	Extinction correction: none

### Special details

**Table:**

Geometry. All e.s.d.'s (except the e.s.d. in the dihedral angle between two l.s. planes) are estimated using the full covariance matrix. The cell e.s.d.'s are taken into account individually in the estimation of e.s.d.'s in distances, angles and torsion angles; correlations between e.s.d.'s in cell parameters are only used when they are defined by crystal symmetry. An approximate (isotropic) treatment of cell e.s.d.'s is used for estimating e.s.d.'s involving l.s. planes.
Refinement. Refinement of *F*^2^ against ALL reflections. The weighted *R*-factor *wR* and goodness of fit *S* are based on *F*^2^, conventional *R*-factors *R* are based on *F*, with *F* set to zero for negative *F*^2^. The threshold expression of *F*^2^ > σ(*F*^2^) is used only for calculating *R*-factors(gt) *etc*. and is not relevant to the choice of reflections for refinement. *R*-factors based on *F*^2^ are statistically about twice as large as those based on *F*, and *R*- factors based on ALL data will be even larger.

### Fractional atomic coordinates and isotropic or equivalent isotropic displacement parameters (Å^2^)

**Table:**

	*x*	*y*	*z*	*U*_iso_*/*U*_eq_	
Cu1	0.36691 (10)	0.08450 (10)	0.90150 (9)	0.0298 (3)	
Cl1	0.1132 (2)	0.0034 (2)	1.1136 (2)	0.0345 (5)	
Cl2	0.4814 (2)	−0.1536 (2)	0.9274 (2)	0.0355 (5)	
N1	0.7388 (7)	0.7150 (7)	0.1354 (6)	0.0310 (14)	
N2	0.8962 (6)	0.7124 (6)	0.1260 (6)	0.0301 (14)	
H2	0.9629	0.7922	0.0931	0.036*	
N3	0.5561 (7)	0.1394 (7)	0.6871 (6)	0.0332 (14)	
N4	0.7207 (7)	0.1464 (7)	0.6647 (6)	0.0364 (15)	
H4	0.7525	0.1184	0.7371	0.044*	
C1	0.4005 (9)	0.1972 (9)	0.5280 (8)	0.0386 (18)	
H1A	0.3168	0.1271	0.6123	0.058*	
H1B	0.4246	0.1673	0.4308	0.058*	
H1C	0.3574	0.2989	0.5284	0.058*	
C2	0.5597 (8)	0.1946 (7)	0.5478 (8)	0.0282 (15)	
C3	0.7295 (8)	0.2400 (7)	0.4356 (7)	0.0284 (15)	
C4	0.8263 (9)	0.2018 (8)	0.5166 (8)	0.0343 (17)	
C5	1.0159 (9)	0.2144 (10)	0.4652 (9)	0.045 (2)	
H5A	1.0463	0.3166	0.4655	0.068*	
H5B	1.0764	0.1921	0.3618	0.068*	
H5C	1.0455	0.1425	0.5356	0.068*	
C6	0.7909 (9)	0.3024 (8)	0.2656 (8)	0.0320 (17)	
H6A	0.7166	0.2605	0.2311	0.038*	
H6B	0.9039	0.2644	0.2102	0.038*	
C7	0.8000 (8)	0.4739 (7)	0.2171 (7)	0.0265 (15)	
C8	0.6772 (8)	0.5694 (7)	0.1915 (7)	0.0252 (15)	
C9	0.5023 (9)	0.5253 (8)	0.2174 (9)	0.0382 (18)	
H9A	0.4223	0.5283	0.3234	0.057*	
H9B	0.5027	0.4234	0.1972	0.057*	
H9C	0.4699	0.5959	0.1482	0.057*	
C10	0.9385 (8)	0.5715 (8)	0.1735 (7)	0.0267 (15)	
C11	1.1096 (9)	0.5461 (9)	0.1710 (9)	0.042 (2)	
H11A	1.1906	0.6210	0.0904	0.064*	
H11B	1.1453	0.4451	0.1508	0.064*	
H11C	1.1028	0.5560	0.2701	0.064*	

### Atomic displacement parameters (Å^2^)

**Table:**

	*U*^11^	*U*^22^	*U*^33^	*U*^12^	*U*^13^	*U*^23^
Cu1	0.0340 (5)	0.0250 (5)	0.0293 (5)	0.0015 (4)	−0.0153 (4)	0.0012 (4)
Cl1	0.0322 (9)	0.0351 (11)	0.0354 (10)	−0.0042 (8)	−0.0186 (8)	0.0064 (8)
Cl2	0.0457 (11)	0.0258 (10)	0.0402 (10)	0.0037 (8)	−0.0242 (9)	−0.0050 (8)
N1	0.028 (3)	0.028 (3)	0.033 (3)	−0.001 (3)	−0.013 (3)	0.003 (3)
N2	0.024 (3)	0.024 (3)	0.038 (3)	−0.003 (2)	−0.014 (3)	0.006 (3)
N3	0.033 (3)	0.035 (4)	0.029 (3)	0.008 (3)	−0.015 (3)	0.000 (3)
N4	0.037 (4)	0.043 (4)	0.034 (3)	0.001 (3)	−0.024 (3)	0.002 (3)
C1	0.039 (4)	0.043 (5)	0.036 (4)	0.002 (4)	−0.019 (4)	−0.004 (4)
C2	0.033 (4)	0.019 (3)	0.037 (4)	0.002 (3)	−0.020 (3)	−0.003 (3)
C3	0.035 (4)	0.018 (4)	0.029 (4)	0.004 (3)	−0.014 (3)	−0.001 (3)
C4	0.040 (4)	0.034 (4)	0.028 (4)	0.002 (3)	−0.018 (3)	0.003 (3)
C5	0.035 (4)	0.061 (6)	0.041 (5)	−0.001 (4)	−0.023 (4)	0.002 (4)
C6	0.037 (4)	0.028 (4)	0.029 (4)	0.010 (3)	−0.016 (3)	−0.001 (3)
C7	0.031 (4)	0.023 (4)	0.026 (4)	0.001 (3)	−0.014 (3)	−0.004 (3)
C8	0.029 (4)	0.021 (3)	0.021 (3)	0.004 (3)	−0.009 (3)	0.000 (3)
C9	0.032 (4)	0.031 (4)	0.060 (5)	−0.007 (3)	−0.028 (4)	−0.007 (4)
C10	0.021 (3)	0.033 (4)	0.022 (3)	−0.003 (3)	−0.007 (3)	−0.001 (3)
C11	0.035 (4)	0.047 (5)	0.041 (4)	0.011 (4)	−0.016 (4)	−0.004 (4)

### Geometric parameters (Å, °)

**Table:**

Cu1—N3	1.993 (5)	C3—C4	1.386 (9)
Cu1—N1^i^	2.009 (6)	C3—C6	1.495 (9)
Cu1—Cl1	2.2926 (19)	C4—C5	1.510 (9)
Cu1—Cl2	2.310 (2)	C5—H5A	0.9600
Cu1—Cl2^ii^	2.712 (2)	C5—H5B	0.9600
Cl2—Cu1^ii^	2.712 (2)	C5—H5C	0.9600
N1—N2	1.340 (7)	C6—C7	1.504 (9)
N1—C8	1.346 (8)	C6—H6A	0.9700
N1—Cu1^i^	2.009 (6)	C6—H6B	0.9700
N2—C10	1.343 (8)	C7—C10	1.386 (9)
N2—H2	0.8600	C7—C8	1.414 (9)
N3—C2	1.340 (8)	C8—C9	1.499 (9)
N3—N4	1.362 (7)	C9—H9A	0.9600
N4—C4	1.332 (8)	C9—H9B	0.9600
N4—H4	0.8600	C9—H9C	0.9600
C1—C2	1.489 (9)	C10—C11	1.493 (9)
C1—H1A	0.9600	C11—H11A	0.9600
C1—H1B	0.9600	C11—H11B	0.9600
C1—H1C	0.9600	C11—H11C	0.9600
C2—C3	1.422 (9)		
			
N3—Cu1—N1^i^	88.7 (2)	N4—C4—C5	120.3 (6)
N3—Cu1—Cl1	165.01 (18)	C3—C4—C5	131.7 (6)
N1^i^—Cu1—Cl1	88.83 (16)	C4—C5—H5A	109.5
N3—Cu1—Cl2	89.48 (17)	C4—C5—H5B	109.5
N1^i^—Cu1—Cl2	174.58 (17)	H5A—C5—H5B	109.5
Cl1—Cu1—Cl2	91.58 (7)	C4—C5—H5C	109.5
N3—Cu1—Cl2^ii^	100.44 (18)	H5A—C5—H5C	109.5
N1^i^—Cu1—Cl2^ii^	100.88 (18)	H5B—C5—H5C	109.5
Cl1—Cu1—Cl2^ii^	94.55 (7)	C3—C6—C7	117.1 (6)
Cl2—Cu1—Cl2^ii^	84.47 (7)	C3—C6—H6A	108.0
Cu1—Cl2—Cu1^ii^	95.53 (7)	C7—C6—H6A	108.0
N2—N1—C8	105.5 (5)	C3—C6—H6B	108.0
N2—N1—Cu1^i^	120.4 (4)	C7—C6—H6B	108.0
C8—N1—Cu1^i^	133.3 (5)	H6A—C6—H6B	107.3
N1—N2—C10	112.6 (5)	C10—C7—C8	104.7 (6)
N1—N2—H2	123.7	C10—C7—C6	126.9 (6)
C10—N2—H2	123.7	C8—C7—C6	128.1 (6)
C2—N3—N4	105.8 (5)	N1—C8—C7	110.2 (6)
C2—N3—Cu1	133.1 (5)	N1—C8—C9	121.6 (6)
N4—N3—Cu1	120.4 (4)	C7—C8—C9	128.3 (6)
C4—N4—N3	111.6 (5)	C8—C9—H9A	109.5
C4—N4—H4	124.2	C8—C9—H9B	109.5
N3—N4—H4	124.2	H9A—C9—H9B	109.5
C2—C1—H1A	109.5	C8—C9—H9C	109.5
C2—C1—H1B	109.5	H9A—C9—H9C	109.5
H1A—C1—H1B	109.5	H9B—C9—H9C	109.5
C2—C1—H1C	109.5	N2—C10—C7	106.9 (5)
H1A—C1—H1C	109.5	N2—C10—C11	120.3 (6)
H1B—C1—H1C	109.5	C7—C10—C11	132.8 (7)
N3—C2—C3	110.0 (6)	C10—C11—H11A	109.5
N3—C2—C1	120.3 (6)	C10—C11—H11B	109.5
C3—C2—C1	129.7 (6)	H11A—C11—H11B	109.5
C4—C3—C2	104.5 (6)	C10—C11—H11C	109.5
C4—C3—C6	127.9 (6)	H11A—C11—H11C	109.5
C2—C3—C6	127.5 (6)	H11B—C11—H11C	109.5
N4—C4—C3	108.0 (6)		

Symmetry codes: (i) −*x*+1, −*y*+1, −*z*+1; (ii) −*x*+1, −*y*, −*z*+2.

### Hydrogen-bond geometry (Å, °)

**Table:**

*D*—H···*A*	*D*—H	H···*A*	*D*···*A*	*D*—H···*A*
N2—H2···Cl1^iii^	0.86	2.43	3.242 (6)	157
N4—H4···Cl1^ii^	0.86	2.34	3.172 (6)	164

Symmetry codes: (iii) *x*+1, *y*+1, *z*−1; (ii) −*x*+1, −*y*, −*z*+2.
